# Genetic diversity of *Listeria monocytogenes* strains in ruminant abortion and rhombencephalitis cases in comparison with the natural environment

**DOI:** 10.1186/s12866-019-1676-3

**Published:** 2019-12-18

**Authors:** Bojan Papić, Mateja Pate, Benjamin Félix, Darja Kušar

**Affiliations:** 1 Institute of Microbiology and Parasitology, Veterinary Faculty, University of Ljubljana, SI-1000 Ljubljana, Slovenia; 2 ANSES, French Agency for Food, Environmental and Occupational Health & Safety, Laboratory for Food Safety, European Union Reference Laboratory for Listeria monocytogenes, University of Paris-Est, 94700 Maisons-Alfort, France

**Keywords:** *Listeria monocytogenes*, Listeriosis, Ruminant, Natural environment, PFGE, MLST, Population structure

## Abstract

**Background:**

*Listeria monocytogenes* is the causative agent of listeriosis, a serious disease affecting both animals and humans. Here, multilocus sequence typing (MLST) was used to characterize the genetic diversity of *Listeria monocytogenes* strains isolated from the natural environment and animal clinical cases in Europe. The prevalence of clonal complexes (CCs) obtained was compared according to (*i*) the origin of isolation – clinical cases vs. natural environment – and (*ii*) the clinical form of animal listeriosis – rhombencephalitis vs. abortion. To this aim, two datasets were constructed. The clinical dataset consisted of 350 animal clinical isolates originating from France and Slovenia and supplemented with isolates from Switzerland and Great Britain. The natural environment dataset consisted of 253 isolates from the natural environment originating from Slovenia and supplemented with isolates from nine other European countries.

**Results:**

For the clinical cases, CC1, CC4-CC217 and CC412 were the most prevalent in rhombencephalitis and CC1, CC37 and CC4-CC217 in abortion. The hypervirulent CC1 and CC4-CC217 prevailed in both datasets. These results indicated that livestock is constantly exposed to hypervirulent CCs. CC1 was significantly associated with a clinical origin, whereas CC9, CC29 and CC14 were associated with the natural environment. CC1 was predominant among rhombencephalitis cases both in cattle and small ruminants, and its prevalence did not differ significantly between these two groups. A novel association of CC37 and CC6 with abortion cases was revealed.

**Conclusions:**

Here, we show that CC1 and CC4-CC217 are prevalent in isolates of environmental and animal clinical origin, suggesting that ruminants are frequently exposed to hypervirulent CCs. The presence of CC4 in two mastitis cases calls for further attention due to direct threat to the consumer. We showed several associations between CCs and the origin of isolation or clinical form of listeriosis, e.g. CC37 and CC6 with abortion. This study improves our understanding of the population structure of *L. monocytogenes* isolates from the natural environment and animal clinical cases. Moreover, it provides a basis for future studies aiming to determine the underlying mechanisms of phenotypic traits of interest.

## Background

*Listeria monocytogenes* is a Gram-positive bacterium that is ubiquitously present in the natural environment (e.g. soil, water and decaying vegetation), but can also cause life-threatening infections in humans and animals [1]. Among farm animals, listeriosis primarily affects small ruminants and cattle. Furthermore, ruminants can act as a reservoir, shedding *L. monocytogenes* asymptomatically into the environment through feces [2]. Improperly fermented, low-quality silage is believed to be the major culprit for the animal listeriosis [3]. The natural environment, in particular the immediate ruminant environment (e.g. feed bunk, water through and bedding), is subject to contamination with *L. monocytogenes* by animals and may be an additional source of infection in animals [4].

In animals, listeriosis can manifest itself in different clinical forms, ranging from a localized infection of the udder (mastitis), the eye (keratoconjunctivitis, uveitis) or gastroenteritis to the more severe, invasive forms of listeriosis including septicemia, rhombencephalitis and infection of the gravid uterus, often leading to premature birth or abortion [2, 5]. Of the severe clinical manifestations of listeriosis, rhombencephalitis is the most common and is associated with a high mortality rate, potentially leading to substantial economic losses in the livestock industry [2, 6].

In Slovenia, the surveillance system of animal listeriosis cases is passive and relies on reporting the neurological symptoms of the disease. The average reported incidence rate of listerial rhombencephalitis in ruminants during the 2006–2016 period was 5.3 cases per 100,000 small ruminants and 1.0 case per 100,000 cattle, with no significant increase in recent years [7, 8]. This incidence rate is markedly higher than the incidence rate in humans in Slovenia, which was 0.63–0.87 per 100,000 people in the 2013–2017 period [9]. The prevalence of ruminant listeriosis in Slovenia is most likely underestimated because many diseased animals do not undergo post mortem examination. Furthermore, the success of such passive surveillance system largely depends on farmers’ awareness of the disease and their willingness to contact the veterinarian [2]. For comparison, an extensive active surveillance study carried out in Switzerland revealed that listeriosis is the most common central nervous system disease in adult small ruminants with an incidence rate of 26.3 cases per 100,000 animals [10]. These results give an idea of the underestimation of listeriosis incidence rate by passive surveillance. Furthermore, the surveillance system, which is carried out in Slovenia and Switzerland, relies only on reporting the neurological symptoms of the disease in ruminants and does not consider another important clinical form of listeriosis – infection of the gravid uterus (abortion). Detection of the latter requires targeted research, which is not a part of the usual routine surveillance.

A variety of typing methods have been employed to differentiate *L. monocytogenes* isolates, including *Eco*RI ribotyping, serotyping, multilocus genotyping (MLGT), multilocus variable-number tandem-repeat analysis (MLVA), pulsed-field gel electrophoresis (PFGE), multilocus sequence typing (MLST) and whole-genome sequencing (WGS) [3, 11–14]. Due to largely clonal reproductive nature and high genetic diversity of *L. monocytogenes*, MLST and WGS-based typing such as core genome MLST (cgMLST) have proved particularly suitable for studies on the population structure and epidemiology of *L. monocytogenes* [13–16].

Until now, MLST-based population studies have mainly been conducted on isolates from human clinical cases and food [13, 17–21]. Previous studies have suggested associations of *L. monocytogenes* genotypes with different clinical forms of animal listeriosis, but did not use MLST [22, 23]. Two European studies [24, 25] included MLST data in their analysis; they focused on rhombencephalitis strains and identified CC1 as the most prevalent CC in ruminant listeriosis, having a strong association with this specific pathology.

The first objective of this study was to improve our knowledge on *L. monocytogenes* population structure in animal clinical cases and in the natural environment. For this purpose, two large datasets were constructed. The clinical dataset consisted of strains isolated from animals with different clinical forms of listeriosis – primarily ruminant abortion and rhombencephalitis cases. The second dataset consisted of strains from the natural environment. The rhombencephalitis isolates, the isolates from cases with other pathology and the environmental isolates primarily originated from Slovenia and were obtained within the framework of the passive surveillance. The abortion isolates primarily originated from one French department and were obtained in the framework of a targeted research project. To increase their size, both datasets were supplemented with other recent European isolates from animal pathology and the natural environment. These data were obtained from the literature and publicly available databases. Based on the constructed datasets, the second objective of the present study was to identify statistically significant associations of *L. monocytogenes* CCs and the clinical forms of listeriosis. The third objective of the study was to identify the most prevalent CCs in the natural environment to which animals are constantly exposed, with emphasis on the isolates from animal feed.

## Results

### Prevalence of CCs in the complete animal clinical dataset

**Fig. 1:**
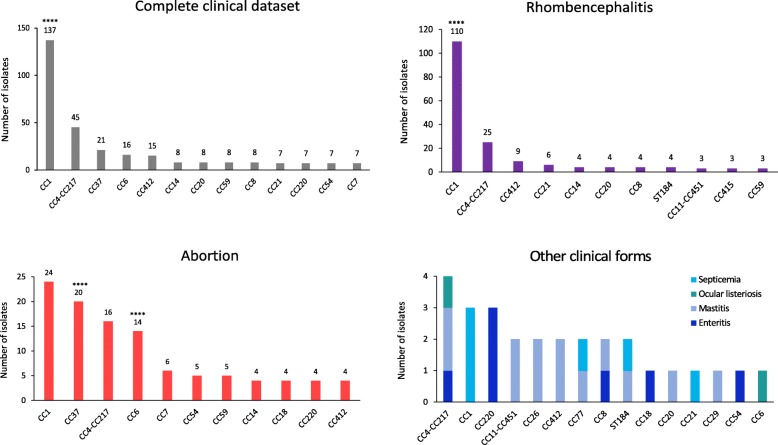
Number of *Listeria monocytogenes* isolates of each clonal complex (CC) in the animal clinical dataset according to the clinical form. The clinical dataset consisted of 350 isolates; the analyzed clinical forms were rhombencephalitis (*n* = 194), abortion (*n* = 128) and other (rare) clinical forms, namely mastitis (*n* = 13), enteritis (*n* = 7), septicemia (*n* = 6) and ocular listeriosis (*n* = 2). Only the most frequent CCs are shown, except in ‘Other clinical forms’. Numbers above the columns indicate the CC frequency. Statistically significant associations (Fisher’s exact test) with a clinical origin (‘All clinical forms’) or a specific clinical form of listeriosis (‘Rhombencephalitis’, ‘Abortion’) are indicated with asterisks. Significance levels: * *p* < 0.05, ** *p* < 0.005, *** *p* < 0.0005, **** *p* < 0.0001

The following CCs were the most prevalent clones in the complete animal clinical dataset (*n* = 350), having frequency of *n* > 10 and comprising 66.9% of the total database: CC1 (137/350; 39.1%), CC4-CC217 (45/350; 12.9%), CC37 (21/350; 6.0%), CC6 (16/350; 4.6%) and CC412 (15/350; 4.3%) (Fig. 1). The percentage of isolates of the eight most frequent CCs (CCs with a frequency of *n* > 10 in any given dataset) in both datasets was compared. CC1 had the highest clinical frequency/environmental frequency ratio of all clones (3.0; Additional file 3: Figure S1). The PFGE profiles (*Asc*I-*Apa*I) of CC4 and CC217 could not be reliably separated because they clustered together according to the 85% similarity rule; therefore, a merged clone CC4-CC217 was constructed for MLST prediction. Considering the results of in silico MLST, the merged CC4-CC217 cluster (*n* = 45) was mainly composed of CC4 isolates (43/45) and a few CC217 isolates (2/45); therefore, the majority of the merged CC4-CC217 isolates most likely belong to CC4.

Among the rhombencephalitis isolates (*n* = 194), CC1 (110/194; 56.7%), CC4-CC217 (25/194; 12.9%) and CC412 (9/194; 4.6%) were the most prevalent CCs, comprising 74.2% of all rhombencephalitis isolates (Fig. 1). In particular, CC1 was the most prevalent CC in all the invasive clinical forms of listeriosis: rhombencephalitis (110/194; 56.7%), abortion (24/128; 18.8%) and septicemia (3/6). The prevalence of CC1 rhombencephalitis isolates in cattle (38/56) and small ruminants (71/137; 51.8%) did not differ significantly (*p* = 0.0546). Among the isolates obtained from abortion cases (*n* = 128), CC1 (24/128; 18.8%), CC37 (20/128; 15.6%) and CC4-CC217 (16/128; 12.5%) prevailed, comprising 46.9% of all abortion isolates (Fig. 1). All other clinical forms of listeriosis (gastroenteritis, mastitis, ocular listeriosis, septicemia) were put into a single group (‘Other clinical forms’, *n* = 28) due to their low frequency and high genetic diversity (Fig. 1). All but one isolate (349/350) had a corresponding lineage defined in the MLST scheme. Lineage I (233/349; 66.8%) was the most prevalent lineage, followed by lineage II (116/349; 33.2%). Lineage III and lineage IV isolates were not detected.

In the French (FR) subset of the animal clinical dataset (*n* = 110), the three most prevalent CCs were CC1 (33/110; 30.0%), CC37 (19/110; 17.3%) and CC4-CC217 (18/110; 16.4%) (Additional file 4: Figure S2). The prevalence of CC1 and CC4-CC217 did not differ significantly from their prevalence in the complete clinical dataset (*p* ≥ 0.0901). Because the FR clinical dataset consisted mostly of abortion isolates (93/110; 84.5%), the prevalence of CCs among the FR abortion isolates was compared with their prevalence among all abortion isolates in the complete clinical dataset. The prevalence of all CCs with the frequency of *n* > 10 (CC1, CC37, CC4-CC217 and CC6) did not differ significantly between the compared groups (*p* ≥ 0.8406). The FR isolates belonged to lineage I (81/110; 73.6%) and lineage II (29/110; 26.4%); lineage III and lineage IV isolates were not detected.

In the Slovenian (SI) subset of the animal clinical dataset (*n* = 55), which was mostly composed of rhombencephalitis isolates (45/55), the three most prevalent clones were CC1 (24/55), CC4-CC217 (7/55) and ST184 (6/55) (Additional file 4: Figure S2). The prevalence of the six most prevalent CCs in the complete animal clinical dataset with a CC frequency of *n* > 10 (CC1, CC4-CC217, CC37, CC6 and CC412) did not differ significantly from their prevalence in the SI subset (*p* ≥ 0.0935). CC1 was significantly over-represented among the SI animal clinical isolates in comparison with the SI environmental isolates (*p* = 0.0083). CC1 also prevailed among the SI rhombencephalitis isolates (23/45). Because the SI dataset consisted mostly of rhombencephalitis isolates (45/55), the prevalence of CCs among the SI rhombencephalitis isolates was compared with their prevalence among all rhombencephalitis isolates in the complete clinical dataset. The prevalence of both CCs with a frequency of *n* > 10 (CC1 and CC4-CC217) did not differ significantly between the compared groups (*p* ≥ 0.5099). All but one isolate (54/55) had a corresponding lineage defined in the MLST scheme. The SI isolates belonged to lineage I (35/54) and lineage II (19/54); lineage III and lineage IV isolates were not detected.

### Prevalence of CCs in the complete natural environment dataset

**Fig. 2:**
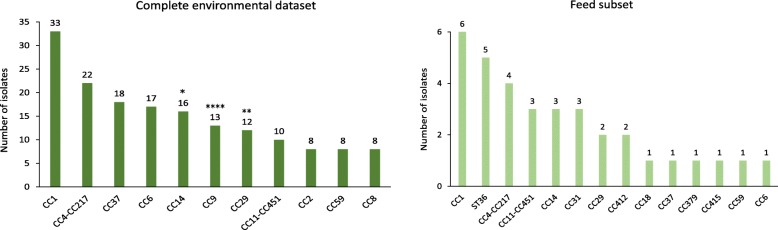
Number of *Listeria monocytogenes* isolates of each clonal complex (CC) in the natural environment dataset, including the animal feed. The environmental dataset (left) consisted of 253 isolates, of which 34 originated from animal feed (right). Only the most frequent CCs are shown, except in ‘Feed’. Numbers above the columns indicate the CC frequency. Statistically significant associations (Fisher’s exact test) with the natural environment (‘Complete environmental dataset’) are indicated with asterisks. Significance levels: * *p* < 0.05, ** *p* < 0.005, *** *p* < 0.0005, **** *p* < 0.0001

The following seven CCs were the most prevalent CCs in the complete natural environment dataset (*n* = 253), having frequency of *n* > 10 and comprising 51.8% of the complete database: CC1 (33/253; 13.0%), CC4-CC217 (22/253; 8.7%), CC37 (18/253; 7.1%), CC6 (17/253; 6.7%), CC14 (16/253; 6.3%), CC9 (13/253; 5.1%) and CC29 (12/253; 4.7%) (Fig. 2). CC9 had the highest environmental frequency/clinical frequency ratio of all clones (18.0; Additional file 3: Figure S1). All isolates had a corresponding lineage defined in the MLST scheme. Lineage II (143/253; 56.5%) was the most prevalent lineage, followed by lineage I (108/253; 42.7%) and lineage III (2/253; 0.8%). Lineage IV isolates were not detected. The most prevalent CCs among the 34 isolates from the animal feed were CC1 (6/34), ST36 (5/34) and CC4-CC217 (4/34) (Fig. 2).

In the SI subset of the environmental dataset (*n* = 40), the most prevalent CC was CC1 (7/40), followed by CC5, CC6, CC29 and CC31 (all 3/40) (Additional file 5: Figure S3). The prevalence of the seven most prevalent CCs in the complete environmental dataset with a CC frequency of *n* > 10 (CC1, CC4-CC217, CC37, CC6, CC14, CC9 and CC29) did not differ significantly from their prevalence in the SI subset of the environmental dataset (*p* ≥ 0.1406). The SI isolates belonged to lineage I (21/40) and lineage II (19/40); lineage III and lineage IV isolates were not detected.

### Association of phylogenetic lineage and CC with the origin of isolation

Lineage I clones were significantly associated with a clinical origin (*p* < 0.0001) and lineage II clones with the natural environment (*p* < 0.0001). CC1 was significantly associated with a clinical origin (*p* < 0.0001) (Fig. 1). On the other hand, CC9 (*p* < 0.0001), CC29 (*p* = 0.0013) and CC14 (*p* = 0.0185) were significantly associated with the natural environment (Fig. 2). In total, 14 isolates belonging to CC29 were present in the constructed datasets. Of these, 12 isolates were obtained from the natural environment, originating from four different European countries (Austria, Italy, Slovenia and Switzerland).

### Association of CCs with the clinical form of listeriosis

No significant difference in the prevalence of the major CCs (CC1, CC4-CC217 and CC37) was identified between small ruminants and cattle for the two major clinical forms of listeriosis (*p* ≥ 0.0738 for abortion and *p* ≥ 0.0546 for rhombencephalitis). Therefore, for the purpose of statistical comparison, the clinical isolates were not stratified according to the animal species but rather according to the clinical form of the disease. Among the isolates of clinical origin, CC1 was significantly (*p* < 0.0001) associated with rhombencephalitis, whereas CC37 (*p* < 0.0001) and CC6 (*p* < 0.0001) were significantly associated with abortion (Fig. 1).

### Taxonomic richness and diversity of *L. monocytogenes* isolates

**Fig. 3:**
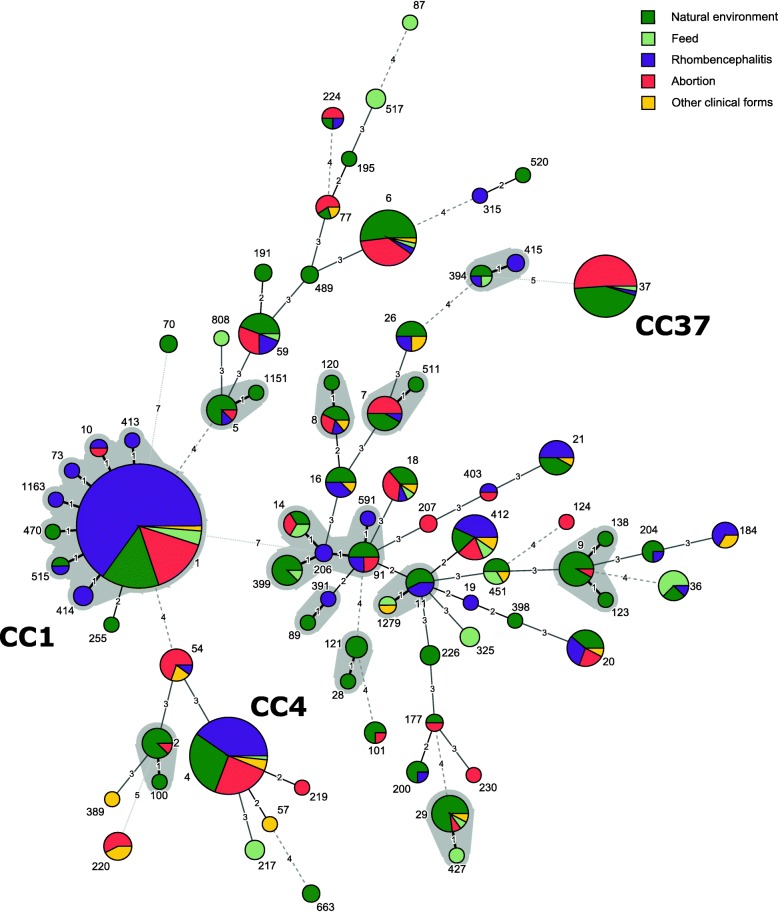
Minimum spanning tree of *Listeria monocytogenes* sequence types (STs) in the clinical and the natural environment dataset. A total of 253 environmental and 350 animal clinical isolates were included into the analysis. Each ST is indicated by a circle whose size reflects the number of isolates, and the STs differing by only one allele are shaded in gray. The numbers on the connecting lines indicate the number of allelic differences in the MLST scheme

When isolates from both datasets were pooled together (*n* = 603), the MLST data showed high genetic diversity (45 different CCs and seven different singleton STs) and non-homogeneous distribution of CCs (Fig. 3). CC1 (170/603; 28.2%), CC4-CC217 (67/603; 11.1%) and CC37 (39/603; 6.5%) were the most prevalent CCs in both datasets, representing 45.8% of all analyzed isolates.

**Fig. 4:**
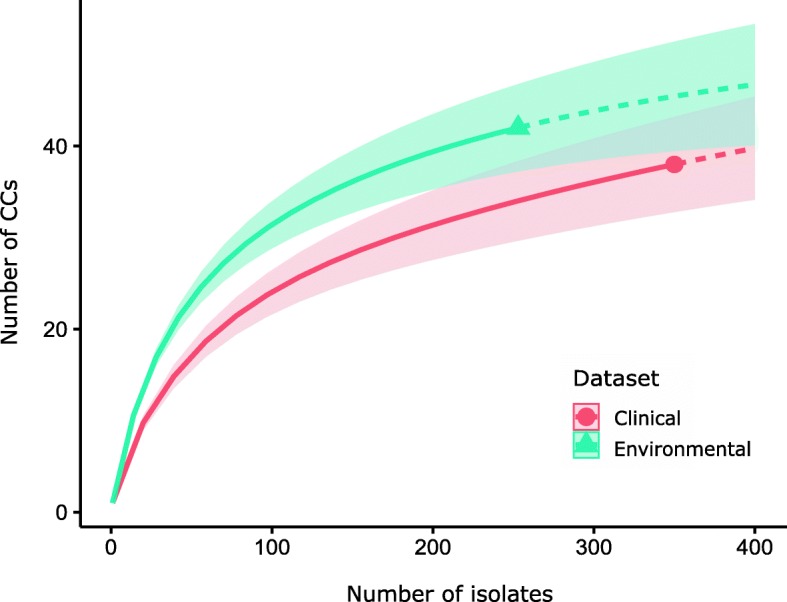
Rarefaction curves showing diversity of *Listeria monocytogenes* clonal complexes (CCs) in the clinical and the natural environment dataset. The solid lines represent the observed accumulation of CCs, and dashed lines represent the extrapolated accumulation. Shaded areas are the 95% confidence intervals

In the animal clinical dataset (*n* = 350), 35 different CCs (of these, six CCs were merged into three combined CCs) and three different singleton STs were identified. The Simpson’s index of diversity was 0.820; 95% confidence interval (CI): 0.783–0.856. In the natural environment dataset (*n* = 253), 36 different CCs (of these, two CCs were merged) and six different singleton STs were identified. The Simpson’s index of diversity was 0.950 (95% CI: 0.941–0.960) and was significantly higher than in the clinical dataset. The rarefaction curves of CCs showed that at the sampling effort of 253 isolates, the observed taxonomic richness is higher in the natural environment dataset, although the 95% CIs overlap. However, a greater sampling effort would be needed in the both analyzed datasets to better describe their diversity because neither of the curves approached the asymptote (Fig. 4). Furthermore, the rarefaction curve of the animal clinical dataset exhibited a steeper curve than the natural environment dataset, indicating that at a higher sampling effort, the clinical isolates may exhibit a similar or greater taxonomic richness in comparison with the environmental isolates.

## Discussion

In the present study, MLST was used to describe the genetic diversity of a panel of 603 *L. monocytogenes* isolates originating from ruminant listeriosis cases and the natural environment. First, 205 isolates originating from France and Slovenia (FR and SI subsets) were typed by MLST. Then, the data obtained was supplemented with the MLST data from 398 European isolates obtained from five publications [15, 24–27] and publicly available databases (NCBI Pathogen Detection browser and Institut Pasteur MLST isolates database). Thus, a large animal clinical and natural environment *L. monocytogenes* dataset were constructed for the MLST-based population analyses, which until now have mainly focused on isolates of human clinical and food origin. The present study is the first report available on diversity of *L. monocytogenes* strains isolated from ruminants in France and Slovenia.

This study showed that distribution of CCs in ruminants, as in humans, is disparate between clinical cases and feed/natural environment (ruminants) or food (humans) (Figs. 1 and 2). In total, 45 CCs and seven singletons were identified; of these, 26 CCs and four singletons were found in both datasets, representing 91.2% of the isolates. A total of 19 CCs and four singletons were unique to a particular dataset. In the present study, CC1, CC4-CC217 and CC37 were the predominant CCs in the natural environment. These CCs were also responsible for the majority of the clinical cases both in small ruminants and in cattle. In humans, the lineage I clones CC1, CC4, CC2 and CC6 were described as clinically associated and hypervirulent. In the food chain, these CCs were frequently encountered, but their prevalence varied between different studies [13, 18, 21].

In the present study, the previously described hypervirulent clones CC1, CC4 and CC6 were among the four most prevalent CCs in both analyzed datasets, which is in accordance with the well-established association between feed or immediate ruminant environment and listeriosis in ruminants [3, 4]. Although the *L. monocytogenes* isolates typed in the present study were regionally limited to the French department *Ille-et-Vilaine* (35) and Slovenia and different clinical forms of listeriosis were prevalent in SI and FR subsets, a similar distribution of major CCs was also observed in both complete datasets (clinical and environmental) consisting of 11 European countries. Further studies are currently undertaken in Europe within the framework of EU One Health European Joint Programme ListAdapt to confirm the data presented herein on a larger European-wide scale [28].

In this study, CC1 was over-represented among ruminant clinical cases and significantly associated with rhombencephalitis, whereas CC9, CC29 and CC14 were significantly associated with the natural environment. A similar association of CC1 with a clinical origin was reported in humans [21]. Conversely, CC9 and CC121 are the two most abundant CCs among food isolates [13, 18]. These CCs were rarely responsible for clinical cases, often associated with patients with immunosuppressive comorbidities and described as hypovirulent in a humanized mouse model [19, 21, 29]. Similar to the food-associated clones CC9 and CC121, CC29 should be screened for the presence of virulence genes and assessed for its virulence potential in animals to explain why it is frequent in the natural environment while rarely responsible for animal listeriosis. Interestingly, CC29 has been rarely reported from the food chain in France and in Europe [21, 30]. This suggests that, in contrast to CC9 and CC121, CC29 is a poor colonizer of the food processing environment. In addition, differences in ecological niche preference may also account for uneven distribution of different CCs. In the present study, the environmental CC29 isolates originated from several European countries. This further confirms the significance of the results and the adaptation of CC29 to the natural environment. The high prevalence of this CC in the natural environment is in congruence with an American study on *L. monocytogenes* in bulk tank milk, in which CC29 was frequently detected in a dairy farm environment [31].

Although a significant association was observed between CC14 and the natural environment, the analyzed CC14 isolates (*n* = 24) belonged to five different STs (ST14, ST91, ST206, ST399 and ST591). Previous studies showed CC14 isolates with indistinguishable PFGE profile belong to different STs, which are genetically relatively distant [21], unpublished data]. Thus, different STs of the same CC may exhibit different ecophysiology, suggesting that in such genetically diverse *L. monocytogenes* groups, further studies should focus on ST rather than CC classification.

The hypervirulent clones CC1 and CC4-CC217 prevailed in both datasets, which confirms the hypothesis that the farm environment represents an important reservoir for *L. monocytogenes*, including the strains with an increased propensity to cause disease in humans [3, 23, 30, 32]. CC1 has also been reported as one of the most prevalent CCs among human clinical isolates in France [21] and Poland [19]. Further comparative genomics studies are needed to investigate whether the same strains are able to cause disease in humans and animals.

In the present study, CC1 was the most prevalent CC among the animal clinical isolates in Slovenia, France and the supplementary European dataset. These results are in accordance with the results of European studies [24, 25], which identified CC1 as the most prevalent CC in ruminant listeriosis. This CC also had a strong association with bovine rhombencephalitis, suggesting its increased neurotropism. However, in contrast to the previously published study [25], we did not observe significant over-representation of CC1 in bovine rhombencephalitis in comparison with small ruminants. CC1 prevailed in all invasive clinical forms of listeriosis (abortion, rhombencephalitis and septicemia), further confirming its hypervirulent nature.

The observed high prevalence of previously reported hypervirulent clones CC4 and CC6 among the animal clinical isolates was similar to that observed in humans [19, 25, 29, 33]. The hypervirulent clone CC4 was reported to have increased cerebral and placental tropism [21], which is in accordance with the results of the present study, showing CC4-CC217 as the second most frequent CC both in rhombencephalitis and abortion. Interestingly, CC4 isolates were detected in all of the inspected animal clinical forms. Among mastitis cases, two isolates belonged to CC4. This is of particular interest as excretion of *L. monocytogenes* in raw milk due to (usually subclinical) listerial mastitis presents a direct threat to consumers or suckling animals. The presence of the hypervirulent clones CC1, CC4 and CC6 in milk and milk products has been previously reported [18, 31].

Due to the low number of isolates obtained from animals with rare forms of listeriosis, the prevalence of different CCs in these cases could not be reliably estimated in the present study. However, the observed high genetic diversity of CCs suggests that other risk factors such as feedstuff quality and storage, farm management practices, animal health and hygiene are the major contributors to the disease in these cases, rather than the virulence of the pathogen [34].

CC2, another previously reported hypervirulent CC, was poorly represented in the analyzed European-wide datasets of *L. monocytogenes* isolates. Its distribution did not differ significantly between the animal clinical (1/350; 0.3%) and natural environment (8/253; 3.2%) datasets. This CC was also not encountered among the SI and FR clinical and environmental isolates typed in the present study. Similarly, low prevalence of CC2 has been previously reported in isolates from the farm environment and animal listeriosis cases [25]. These results contrast with its high prevalence observed among the human clinical isolates [19, 21] and suggests CC2 is not well adapted to the farm environment.

We identified CC6 as an abortion-associated CC. Interestingly, this CC has not been reported as abortion-associated in humans [21], which suggests the existence of abortion-associated CCs that are adapted to non-primates. Moreover, the lineage II clone CC37 was also abortion-associated. In the present study, CC37 was the third most prevalent CC among the animal clinical isolates. Such a high prevalence of CC37 has not yet been reported among the animal or human clinical isolates [19, 21, 25]. This observation needs further confirmation by extended sampling as in the present study, the majority of the abortion isolates were isolated from one French region. However, in several studies [15, 18, 25], CC37 was observed in milk samples, and in the previously published study [31], it was reported as the second most prevalent lineage II clone in milk samples. Therefore, previous findings suggest that it may be adapted to the dairy farm environment and may persist for a long period outside the animal host. In general, the underlying molecular mechanisms affecting tropism remain poorly understood. A recently described LIPI-4 gene cluster is involved in the hypervirulence of CC4, a clone that shows tropism for brain tissue and gravid uterus in a humanized mouse model [21]. Internalin F (InlF) is involved in colonization of the brain in mice [35]. Moreover, a novel virulence factor internalin P is critical for placental infection and thus strongly associated with abortion [36, 37]. Our results suggest that future comparative genomics studies, focusing on CC6 and CC37, may lead to a discovery of candidate genes and/or gene variants presumably involved in increased placental tropism.

In correspondence to the previous findings [25], the lineage II clone CC412 was among the five most prevalent CCs in the animal clinical dataset, although it was not significantly over-represented among the animal clinical isolates in comparison with the natural environment dataset. This CC was also encountered among the FR animal clinical isolates (2/110; 1.8%), but absent from the SI animal clinical isolates. Contrary to its high prevalence among animal clinical isolates, it was not found among the most prevalent CCs in the human clinical isolates in France [21] and Poland [19], which may suggest its adaptation to non-primate animal hosts.

In the present study, CC1 was the most prevalent CC among the isolates from animal feed (in particular, silage), followed by ST36 and CC4-CC217. This finding, in addition to the generally high prevalence of CC1 and CC4-CC217 in the natural environment dataset, suggests that animals are mostly exposed to the hypervirulent CCs such as CC1 and CC4.

Several studies have shown that farm-level management and hygiene practices are associated with the prevalence of *L. monocytogenes* [2, 34, 38–40]. Furthermore, better understanding of the occurrence, ecology and contamination routes of *L. monocytogenes* in the farm environment can help to implement improved control measures to limit the disease burden in animals and the introduction of *L. monocytogenes* into the food chain. In the present study, CC1, CC4-CC217, CC37 and CC6 were the most prevalent CCs in the natural environment dataset. These results underline the risk of ruminant contamination from the natural/farm environment.

In the present study and in the previously published European study [25], CC7 and CC14 were observed both in the clinical and the natural environment dataset, but were not among the three most prevalent clones. In contrast, CC7 (7/46) and CC14 (5/46) were reported as the most prevalent CCs among the animal clinical isolates from the USA [41]. A high prevalence of CC7 at the dairy farm level in the USA was also reported [31]. This discrepancy may result from a different number of analyzed isolates, differences in the population structure of *L. monocytogenes* between continents as well as different monitoring and sampling criteria. Further studies of prevalence of *L. monocytogenes* in Europe are needed. In general, the estimated prevalence of CCs should be interpreted with caution in small datasets, in isolates from a limited geographical area or datasets with epidemiological duplicates. In the present study, the FR and SI subsets were de-duplicated.

Interestingly, 6/55 animal clinical isolates from Slovenia belonged to singleton ST184 (lineage II). This CC was not present among the environmental SI isolates and the remaining isolates from the analyzed datasets and was absent from the Institut Pasteur MLST isolates database at the time of writing. Furthermore, a single ST184 human clinical isolate from USA (NCBI BioSample accession number SAMN03277635) was identified in the NCBI Pathogen Detection system. These findings suggest ST184 is a rare clone with a higher prevalence in Slovenia when compared with other countries, which may be adapted to animal hosts and may have a propensity to cause disease; however, further large-scale studies are needed to confirm this finding. Such regional heterogeneity in the prevalence of certain *L. monocytogenes* CCs has already been reported [41–44].

## Conclusions

In conclusion, we show that CC1 and CC4, previously described as the hypervirulent CCs, were the most prevalent CCs among the isolates of both analyzed origins (animal clinical cases and the natural environment). CC1 was rhombencephalitis-associated, whereas CC6 and CC37 were abortion-associated. CC9, CC14 and CC29 were associated with the natural environment. Our results suggest that animals are mostly exposed to hypervirulent CCs (CC1 and CC4) and that the CC distribution in the natural environment differs from the distribution observed in food. We confirmed the non-homogeneous distribution of *L. monocytogenes* CCs across different origins of isolation (animal clinical vs. environmental) and across different clinical forms of listeriosis (abortion vs. rhombencephalitis). Due to the high genetic diversity and ubiquitous nature of *L. monocytogenes*, a large number of isolates is needed to accurately describe its diversity in different environments. To the best of our knowledge, this is the largest study on the prevalence of *L. monocytogenes* CCs in different clinical forms of listeriosis in animals and in the natural environment in Europe. Furthermore, such large-scale prevalence studies represent a basis for the future studies addressing the virulence and tropism of different *L. monocytogenes* CCs, i.e. studies on the discovery of genetic traits that are involved in adaptation to a specific ecological niche (e.g. animal hosts and environmental conditions) and tissue tropism (e.g. cerebral and placental tropism).

## Materials and methods

### Construction of datasets

In the present study, two large datasets of *L. monocytogenes* isolates of different origin (animal clinical, environmental) were constructed. Clinical isolates were defined here as isolates originating from confirmed cases of animal listeriosis. The definitions of clinical forms of listeriosis were harmonized with those previously described [25, 45, 46]. Abortion cases were defined as isolation of *L. monocytogenes* from the placenta and/or the fetus. Gastroenteritis cases were defined as cases with diarrhea and neutrophilic gastroenteritis, in which *L. monocytogenes* was isolated from the gastrointestinal content. Mastitis cases were defined as isolation of *L. monocytogenes* from the udder quarter. Ocular listeriosis cases were defined as isolation of *L. monocytogenes* from the eye and the presence of ocular inflammation. Rhombencephalitis cases were defined as isolation of *L. monocytogenes* from the brainstem of animals with neurological signs and presence of pathognomonic changes in the brain tissue, confirmed by histopathology. Septicemia cases were defined as isolation of *L. monocytogenes* from visceral organs. The natural environment dataset included isolates that originated from the natural environment (including animal feed), excluding isolates from food, food-associated units or confirmed animal listeriosis cases. Both datasets included isolates that had not been pre-selected according to their typing data and neither of the panels included epidemiological duplicates; these were defined as isolates sharing indistinguishable PFGE profiles, originating from the same animal outbreak or farm environment from the same sampling. To increase the number of isolates in both datasets, a total of 205 French (FR) and Slovenian (SI) isolates originating from the period 2008–2018 were supplemented with additional isolates and corresponding metadata originating from scientific publications and public web databases – *Listeria* Pasteur MLST [47] and NCBI Pathogen Detection system [48]. To ensure the consistency of the datasets, only isolates obtained during the 1990–2018 period and originating from Europe were included. Only the isolates with an assigned CC as well as year, country and origin of isolation were included in the datasets. Hypo- and hypervirulent CCs were defined as previously described; namely, CC9 and CC121 were defined as hypovirulent, whereas CC1, CC2, CC4 and CC6 as hypervirulent [21]. The complete datasets including the metadata associated with the isolates can be found in the Additional file 1: Table S1 and Additional file 2: Table S2.

### Animal clinical dataset

In total, the animal clinical dataset consisted of 350 isolates originating from cases with a well-defined clinical form of listeriosis, defined above. The isolates were classified according to their associated clinical form: abortion, gastroenteritis, ocular listeriosis, rhombencephalitis or septicemia. The SI subset comprised 55 clinical isolates that were obtained during the 2008–2018 period as part of national disease surveillance activities, which included brain samples from ruminants with clinical neurological signs, or during routine monitoring of raw milk. The SI isolates originated from ovine (*n* = 21), bovine (*n* = 19), caprine (*n* = 14) and cervine (*n* = 1) cases. The FR subset of animal clinical isolates consisted of 110 isolates obtained from a prophylactic study including the systematic investigation of causes of abortion in ruminants from 107 farms. This study was conducted by private veterinary laboratories during the 2010–2015 period in the French department of *Ille-et-Vilaine* (35) within the framework of the OSCAR program [49]. The FR isolates originated from bovine (*n* = 95), caprine (*n* = 5), ovine (*n* = 4), cervine (*n* = 1) and leporine (*n* = 1) cases. This dataset was supplemented with 185 isolates and their associated metadata (MLST type and clinical form of listeriosis) from Switzerland and Great Britain [24, 25]. The dataset included 194 rhombencephalitis isolates, 128 abortion isolates and 28 isolates associated with other clinical forms.

### Natural environment dataset

In total, the natural environment dataset consisted of 253 isolates. It comprised 40 SI isolates originating from the natural environment that were obtained as part of national research projects that investigated the prevalence of *L. monocytogenes* in different natural environments during the 2008–2018 period. The SI isolates originated from feed, manure, milking equipment, water or soil and feces from apparently healthy ruminants. This dataset was supplemented with 191 isolates and their associated metadata (source of isolation, MLST type) from Austria, the Czech Republic, Finland, Germany, Great Britain, Greece, Italy, Portugal and Switzerland [15, 25–27].

### PFGE typing (FR and SI subsets)

A total of 95 isolates (55 clinical and 40 environmental isolates) from the SI subset and 110 animal clinical isolates from the FR subset were PFGE-typed according to the method recommended by the EURL *Lm* [50]. Briefly, genomic DNA of *L. monocytogenes* was digested with *Apa*I and *Asc*I restriction endonucleases and the obtained fragments were separated using the CHEF-DR II system (Bio-Rad, USA) according to the recommended electrophoretic protocol. PFGE profiles were analyzed using the BioNumerics v7.6.2 software (Applied Maths, Belgium).

### WGS and in silico MLST (FR and SI subsets)

Among the 205 isolates from the FR and SI subsets, 78 isolates (26 SI and 52 FR isolates) underwent WGS. Of these, eight isolates were sequenced on an Ion Torrent PGM System (Thermo Fisher Scientific, USA), whereas 70 isolates were sequenced on an Illumina NextSeq500 System (USA) due to its higher throughput. Highly concordant results for both technologies have been previously shown with regard to bacterial typing [51]. WGS data were used to determine the affiliated MLST type. Reads were assembled into contigs using SPAdes v3.11.1 [52]. Contigs were used as an input for the in silico generation of MLST type according to the Institut Pasteur *Listeria* MLST scheme [47]. The curation of novel MLST types was kindly performed by database curators at the Institut Pasteur (France).

### Mapping MLST/PFGE clusters

Among the 205 isolates from the FR and SI subset of the constructed datasets, 127 isolates were not typed using WGS. To obtain the CC affiliation of these isolates, their CC was mapped to their PFGE profiles as described previously [18]. The latter study demonstrated the usefulness of a mapping method based on a correlation between 85% similarity PFGE clusters (combined *Asc*I-*Apa*I profiles) and CC. The method uses the high concordance between PFGE clusters and corresponding CCs (average adjusted Wallace index of 0.92). Briefly, a reference panel of 396 isolates typed with PFGE and MLST was used and imported into BioNumerics as a bundle [13, 18]. A combined *Asc*I-*Apa*I dendrogram of the 396 reference isolates and all the analyzed isolates was constructed using the following parameters: unweighted pair group method with arithmetic average (UPGMA), similarity calculated using the Dice coefficient, tolerance, and optimization set to 1%. The isolates sharing ≥85% similarity with the reference isolates of a known MLST type were considered as belonging to the same CC. When two clusters were associated with the same CC, the clusters were merged. When several isolates from different CCs were part of the same cluster, the CCs were merged (e.g. clone CC4-CC217). When the isolates did not share PFGE clusters with any of the isolates typed by MLST, CC was not assigned [18]. Isolates without an assigned MLST type were excluded from the analysis.

### Statistics

Statistical analyses were performed using GraphPad Prism v6.01 (GraphPad Software, USA). All associations of the CCs or phylogenetic lineages with their corresponding traits (animal species, clinical or environmental origin) were tested for their significance using Fisher’s exact test. Therefore, association with a given trait did not depend exclusively on CC prevalence per se. A statistical significance was determined at *p* < 0.05. The statistical analysis was confined to the CCs that showed a frequency of *n* > 10 in any given dataset.

### Taxonomic richness and diversity

The minimum spanning tree of *L. monocytogenes* STs was constructed using BioNumerics v7.6.2 software (Applied Maths, Belgium). For this purpose, the Institut Pasteur *Listeria* MLST scheme [47] was imported into the software. When only the corresponding CC (but not the ST) of the isolate was known, the predicted founder ST of a given CC was used (e.g. ST1 allele combination for CC1). Therefore, the ST designation of the isolates that were typed solely using PFGE is hypothetical. The Comparing Partitions online calculator [53] was used to calculate the Simpson’s index of diversity (1–D), measuring the probability that two isolates randomly selected from a population belong to different types. Rarefaction curves were used to estimate species richness of subsamples drawn from a collection, plotted against the size of subsample; these were obtained with the iNEXT v2.0.17 (https://cran.r-project.org/web/packages/iNEXT/) and ggplot2 v3.0.0 (https://cran.r-project.org/web/packages/ggplot2/) package for R.

## Supplementary information

**Additional file 1: Table S1.** A complete database of *Listeria monocytogenes* isolates from the animal clinical cases.

**Additional file 2: Table S2.** A complete database of *Listeria monocytogenes* isolates from the natural environment.

**Additional file 3:** **Figure S1.** Percentage of *Listeria monocytogenes* isolates of each clonal complex (CC) according to the origin of isolation. The percentage of isolates of each CC in the clinical dataset (*y* axis) was plotted against the percentage of isolates of each CC in the natural environment dataset (*x* axis). The 11 most common CCs in the combined clinical and environmental dataset are shown, representing 85.3% of all isolates (*n* = 603); each CC is indicated by a circle whose size reflects the number of isolates. Lineage I was significantly associated with a clinical origin (*p* < 0.0001) and lineage II with the natural environment (*p* < 0.0001). CCs that were significantly associated with their origin of isolation are shown in bold: CC1 was significantly (*p* < 0.0001) associated with a clinical origin, whereas CC9 (*p* < 0.0001), CC29 (*p* = 0.0013) and CC14 (*p* = 0.0185) were significantly associated with the natural environment.

**Additional file 4:** **Figure S2.** Number of *Listeria monocytogenes* isolates of each clonal complex (CC) in the animal clinical dataset according to the clinical form of listeriosis. The complete animal clinical dataset (*n* = 350) consisted of the French (FR) subset (*n* = 110), the Slovenian (SI) subset (*n* = 55) and the supplementary European dataset (*n* = 185). Only the most frequent CCs are shown, except for the SI subset.

**Additional file 5:** **Figure S3.** Number of *Listeria monocytogenes* isolates of each clonal complex (CC) in the complete natural environment dataset (*n* = 253) and the Slovenian (SI) subset of the natural environment dataset (*n* = 40). CC9 (*p* < 0.0001), CC29 (*p* = 0.0013) and CC14 (*p* = 0.0185) were significantly associated with the natural environment. Only the most frequent CCs are shown.

## Data Availability

All data generated or analyzed during this study are included in this published article and its supplementary information files.

## Abbreviations

CC: clonal complex
MLST: multilocus sequence typing
PFGE: pulsed-field gel electrophoresis
ST: sequence type
WGS: whole-genome sequencing
