# Supplementary figures and images for: Genetic diversity of Listeria monocytogenes strains in ruminant abortion and rhombencephalitis cases in comparison with the natural environment
Source: BMC Microbiol. 2019 Dec 18;19:299. doi: 10.1186/s12866-019-1676-3 (PMC6918561; doi:10.1186/s12866-019-1676-3)

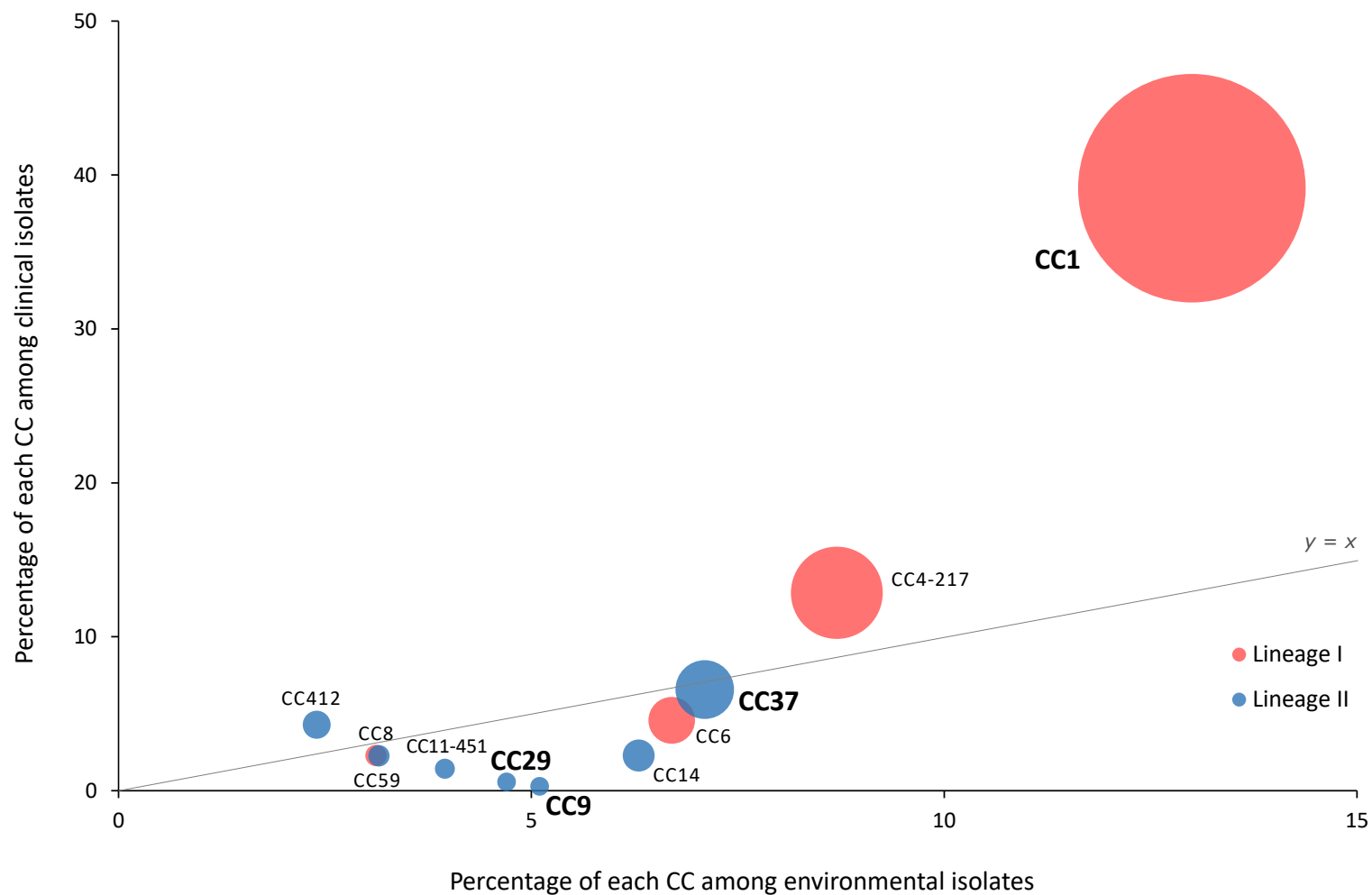

Supplement: Supplementary file 3 — Additional file 3: Figure S1. Percentage of Listeria monocytogenes isolates of each clonal complex (CC) according to the origin of isolation. The percentage of isolates of each CC in the clinical dataset (y axis) was plotted against the percentage of isolates of each CC in the natural environment dataset (x axis). The 11 most common CCs in the combined clinical and environmental dataset are shown, representing 85.3% of all isolates (n = 603); each CC is indicated by a circle whose size reflects the number of isolates. Lineage I was significantly associated with a clinical origin (p < 0.0001) and lineage II with the natural environment (p < 0.0001). CCs that were significantly associated with their origin of isolation are shown in bold: CC1 was significantly (p < 0.0001) associated with a clinical origin, whereas CC9 (p < 0.0001), CC29 (p = 0.0013) and CC14 (p = 0.0185) were significantly associated with the natural environment. [file 12866_2019_1676_MOESM3_ESM.pdf]

Complete environmental dataset

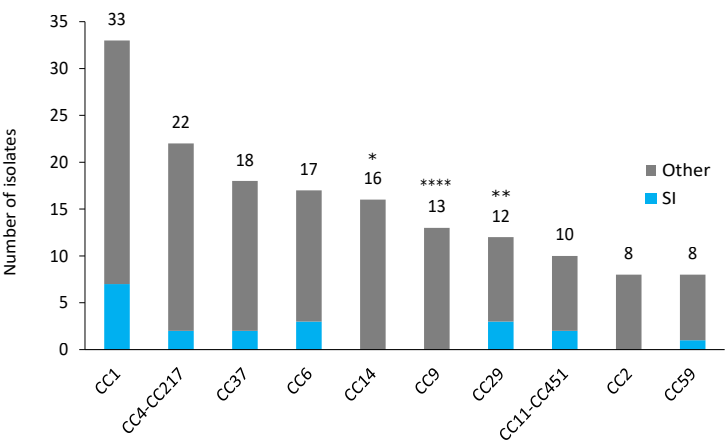

SI subset

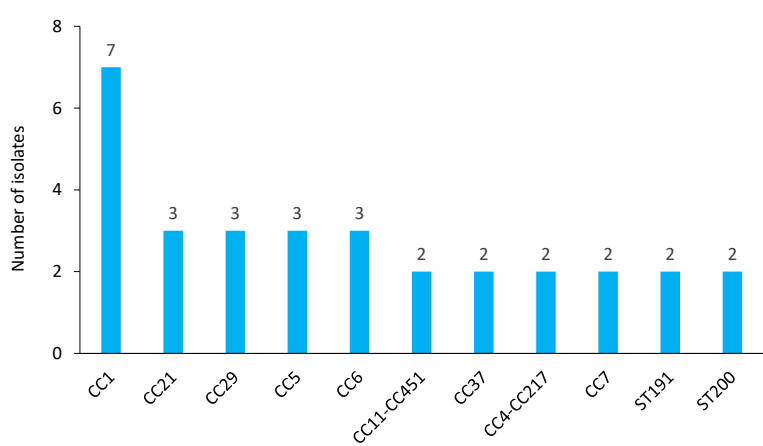

Supplement: Supplementary file 5 — Additional file 5: Figure S3. Number of Listeria monocytogenes isolates of each clonal complex (CC) in the complete natural environment dataset (n = 253) and the Slovenian (SI) subset of the natural environment dataset (n = 40). CC9 (p < 0.0001), CC29 (p = 0.0013) and CC14 (p = 0.0185) were significantly associated with the natural environment. Only the most frequent CCs are shown. [file 12866_2019_1676_MOESM5_ESM.pdf]
