# Supplementary material for: Genetic diversity of Listeria monocytogenes strains in ruminant abortion and rhombencephalitis cases in comparison with the natural environment
Source: BMC Microbiol. 2019 Dec 18;19:299. doi: 10.1186/s12866-019-1676-3 (PMC6918561; doi:10.1186/s12866-019-1676-3)
Supplement: Supplementary file 4 — Additional file 4: Figure S2. Number of Listeria monocytogenes isolates of each clonal complex (CC) in the animal clinical dataset according to the clinical form of listeriosis. The complete animal clinical dataset (n = 350) consisted of the French (FR) subset (n = 110), the Slovenian (SI) subset (n = 55) and the supplementary European dataset (n = 185). Only the most frequent CCs are shown, except for the SI subset. [file 12866_2019_1676_MOESM4_ESM.pdf]

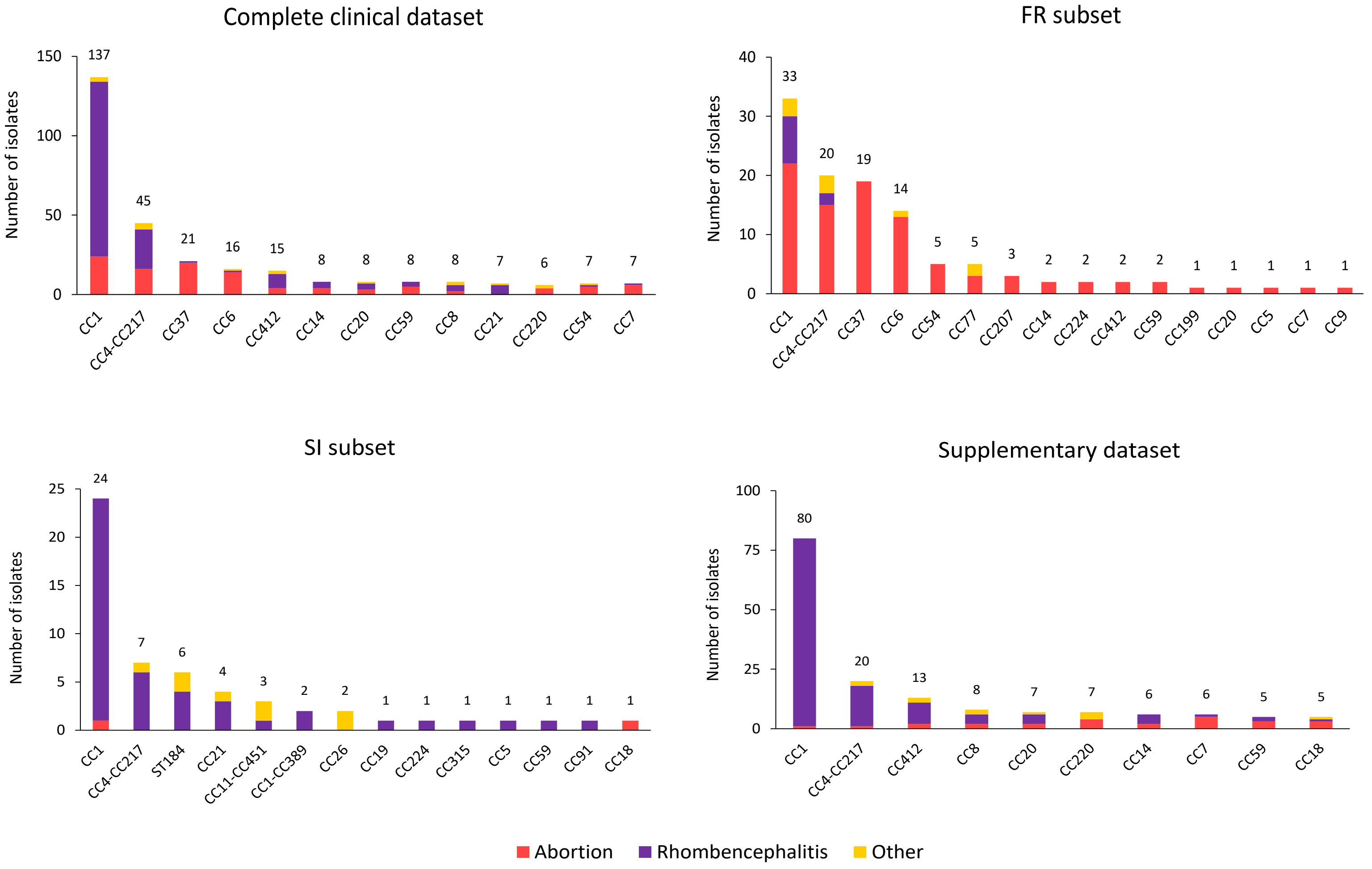

Complete clinical dataset
Number of isolates
150
100
50
0
137
45
21
16
15
8
8
8
8
7
6
7
7
CC1
CC4-CC217
CC37
CC6
CC412
CC14
CC20
CC59
CC8
CC21
CC220
CC54
CC7
FR subset
Number of isolates
40
30
20
10
0
33
20
19
14
5
5
3
2
2
2
2
1
1
1
1
1
CC1
CC4-CC217
CC37
CC6
CC54
CC77
CC207
CC14
CC224
CC412
CC59
CC199
CC20
CC5
CC7
CC9
SI subset
Number of isolates
25
20
15
10
5
0
24
7
6
4
3
2
2
1
1
1
1
1
1
1
CC1
CC4-CC217
ST184
CC21
CC11-CC451
CC1-CC389
CC26
CC19
CC224
CC315
CC5
CC59
CC91
CC18
Supplementary dataset
Number of isolates
100
75
50
25
0
80
20
13
8
7
7
6
6
5
5
CC1
CC4-CC217
CC412
CC8
CC20
CC220
CC14
CC7
CC59
CC18
Abortion
Rhombencephalitis
Other
